# Residential green space and pathways to term birth weight in the Canadian Healthy Infant Longitudinal Development (CHILD) Study

**DOI:** 10.1186/s12942-018-0160-x

**Published:** 2018-12-04

**Authors:** Leanne Cusack, Hind Sbihi, Andrew Larkin, Angela Chow, Jeffrey R. Brook, Theo Moraes, Piush J. Mandhane, Allan B. Becker, Meghan B. Azad, Padmaja Subbarao, Anita Kozyrskyj, Tim K. Takaro, Malcolm R. Sears, Stuart E. Turvey, Perry Hystad, the CHILD Study Investigators, P. Subbarao, S. E. Turvey, M. R. Sears, S. S. Anand, M. B. Azad, A. B. Becker, A. D. Befus, M. Brauer, J. R. Brook, E. Chen, M. M. Cyr, D. Daley, S. D. Dell, J. A. Denburg, Q. L. Duan, T. Eiwegger, H. Grasemann, K. HayGlass, R. G. Hegele, D. L. Holness, P. Hystad, M. Kobor, T. R. Kollmann, A. L. Kozyrskyj, W. Y. W. Lou, J. Macri, P. J. Mandhane, G. Miller, T. J. Moraes, P. Paré, C. Ramsey, F. Ratjen, A. Sandford, J. A. Scott, J. Scott, F. Silverman, J. Scott, E. Simons, T. Takaro, S. J. Tebbutt, T. To

**Affiliations:** 1 Oregon State University, Corvallis, OR USA; 2 University of British Columbia, Vancouver, BC Canada; 3 Indiana University, Bloomington, IN USA; 4 University of Toronto, Toronto, ON Canada; 5 Hospital for Sick Children, Toronto, ON Canada; 6 University of Alberta, Edmonton, AB Canada; 7 University of Manitoba, Winnipeg, MB Canada; 8 Hospital for Sick Children, Toronto, ON Canada; 9 Simon Fraser University, Burnaby, BC Canada; 10 McMaster University, Hamilton, ON Canada; 11 BC Children’s Hospital, Vancouver, BC Canada; 12 College of Public Health and Human Sciences, Oregon State University, 2520 SW Campus Way, Corvallis, OR 97331 USA

**Keywords:** Green space, Nature, Built environment, Birth weight, Birth outcomes

## Abstract

**Background:**

A growing number of studies observe associations between the amount of green space around a mother’s home and positive birth outcomes; however, the robustness of this association and potential pathways of action remain unclear.

**Objectives:**

To examine associations between mother’s residential green space and term birth weight within the Canadian Healthy Infant Longitudinal Development (CHILD) study and examine specific hypothesized pathways.

**Methods:**

We examined 2510 births located in Vancouver, Edmonton, Winnipeg, and Toronto Canada. Green space was estimated around mother’s residences during pregnancy using Landsat 30 m normalized difference vegetation index (NDVI). We examined hypothesized pathways of: (1) reduction of environmental exposure; (2) built environment features promoting physical activity; (3) psychosocial conditions; and (4) psychological influences. Linear regression was used to assess associations between green space and term birth weight adjusting first for a comprehensive set of confounding factors and then incrementally for pathway variables.

**Results:**

Fully adjusted models showed non-statistically significant increases in term birth weight with increasing green space. For example, a 0.1 increase in NDVI within 500 m was associated with a 21.5 g (95% CI − 4.6, 47.7) increase in term birth weight. Associations varied by city and were most robust for high-density locations. For the two largest cities (Vancouver and Toronto), we observed an increase in birth weight of 41.2 g (95% CI 7.8, 74.6) for a 0.1 increase in NDVI within 500 m. We did not observe substantial reductions in the green space effect on birth weight when adjusting for pathway variables.

**Conclusion:**

Our results highlight the need to further characterize the interactions between green space, urban density and climate related factors as well as the pathways linking residential green space to birth outcomes.

## Introduction

Proximity to green spaces has been linked to positive health outcomes. An increased amount of green space around a woman’s home during pregnancy has been associated with a reduced risk of delivering a baby that is small for gestational age [1], lower rates of pre-term birth [2] and higher birth weight at term [3–6]. Importantly, the pathways through which green space may influence these birth outcomes remain unclear [7]. A number of different pathways have been hypothesized, including: (1) the reduction of harmful environmental exposures, including air pollution and noise; (2) providing space for increased utilitarian and recreational physical activity; (3) providing a setting for positive psychosocial influences; and (4) through directly reducing psychological stress and depression [8].

Most green space and birth outcome studies have not been able to comprehensively examine these hypothesized pathways. For example, a study by our group in Vancouver, Canada was able to examine air pollution, walkability and proximity to parks and found that the addition of these variables did not affect the associations between green space and birth weight [6]. In a large Southern California study [2], different air pollutants had considerable impact on the association between green space and birth weight but a study of four Spanish birth cohorts observed only moderate reductions in the green space birth weight association with the inclusion of NO_2_ air pollution [5]. While studies of green space and other health outcomes have started to examine mediation by different pathways [9, 10], direct evidence for the pathways linking green space exposure during pregnancy to birth outcomes does not exist.

Indirect evidence remains important as it provides support for the potential pathways of influence linking green space to birth outcomes. For instance, motor vehicle transportation not only produces air pollutants and noise, but also changes urban form (e.g., walkability) and access to natural environment and green spaces. Thus, while high levels of exposure to ambient air pollution during pregnancy have been shown to be associated with low birth weight [11–14], vegetated areas have less emission sources and may also improve air quality by filtering NO_2_ and particulate matter (PM_10_) [15]. A growing body of research also demonstrates that physical activity during pregnancy can reduce the risk of having a low birth weight baby [16–19] and there is evidence of a positive association between green space access and increased physical activity [20–22]. More recently, measures such as walkability, also associated with residential green space in certain areas, have been linked to higher levels of physical activity [6, 23]. Parks and green spaces can also facilitate social cohesion by providing a meeting place for people to develop and maintain social interactions [24–26], which have also been linked to improved birth outcomes [27, 28]. In a recent study, green space was associated with decreased depressive symptoms in pregnant women [10], and women with depression during pregnancy are at increased risk for adverse birth outcomes [29]. These intertwined exposures and pathways demonstrate the need to examine the specific pathways that potentially link urban green space to term birth weight.

We leveraged the Canadian Healthy Infant Longitudinal Development (CHILD) study (www.childstudy.ca), to examine associations between mother’s residential green space during pregnancy and term birth weight across four Canadian urban areas (Vancouver, Edmonton, Winnipeg, and Toronto). Given the detailed information collected in CHILD we were able to adjust for multiple potential confounding factors and evaluate a comprehensive set of hypothesized pathways. This study provides the first opportunity to broadly examine how urban green space may influence term birth weight.

## Methods

### CHILD birth cohort

The CHILD Study recruited 3623 pregnant women from the general population across 4 Canadian urban metropolitan areas (Vancouver, British Columbia; Edmonton, Alberta; Winnipeg, Manitoba; and Toronto, Ontario) from 2009 to 2012. After excluding 83 children who were ineligible at birth and 45 families who failed to begin the study, we further restricted our analyses to mothers living in metropolitan areas and with complete residential history and covariate data. Our final sample size included 2510 births. Detailed data collection methods and characteristics of the CHILD Study have been previously described [30, 31]. Briefly, pregnant women aged 18 years or older (19 years or older in Vancouver) and infants born after 34 weeks gestation with no congenital abnormalities were eligible. Due to the gestational length restriction we only examine term birth weight (≥ 37 weeks gestation). Questionnaires collected information on environmental exposures, psychosocial factors, and general health at several time points: recruitment (mean 27 weeks gestation), 36 weeks gestation and birth. Ethics approval for each CHILD center was obtained from local authorized review boards as well as McMaster University and Oregon State University.

### Assessment of residential green space

We measured green space exposure using the average of Landsat 5 30 m normalized difference vegetation index (NDVI) around mothers’ residential addresses during the 9 months of pregnancy. A time-weighted average from all addresses during pregnancy (11.5% of participants moved) was computed for different buffer distances (i.e., 100 m, 250 m, 500 m, 1000 m) around addresses since there is no consensus as to what buffer radius is most representative of green space exposures [8]. NDVI is an indicator of overall greenness based on land surface reflectance of the visible and near infrared parts of spectrum [33], with values ranging from − 1 to 1 with the higher numbers indicating more greenness.

### Assessment of the four pathways hypothesized to link green spaces to birth outcomes

We examined four general pathways that are hypothesized to link green space to birth outcomes. These include: (1) the reduction of harmful environmental exposures, particularly air pollution and noise; (2) providing space for increased utilitarian and recreational physical activity; (3) providing a setting for positive psychosocial influences; and (4) through directly reducing psychological stress and depression [8].

#### Air pollution

We included Nitrogen Dioxide (NO_2_), fine particulate matter (PM_2.5_), and Ozone (O_3_) air pollution in our analyses. NO_2_ is of primary interest since this pollutant demonstrates fine-scale spatial variability that is most likely influenced by residential green space levels [32]. We applied a previously developed national land use regression (LUR) model for NO_2_, which includes satellite NO_2_ estimates and land use variables [33]. The final LUR model, which was further spatially calibrated based on a distance-decay gradient in NO_2_ around major roadways, explained 73% of the variation in annual 2006 national air quality monitoring network measurements of NO_2_. We adjusted these estimates based on ground-level annual average measurements of NO_2_ to match pregnancy years. PM_2.5_ air pollution was assessed using 2010–2013 satellite data at an approximate resolution of 1 × 1 km fused with a chemical transport model and available ground level PM_2.5_ monitoring data [34]. Ozone air pollution was assessed using the 2002–2009 Canadian and Hemispheric Regional Ozone and NO_x_ System (CHRONOS) operational regional air quality forecast model at an approximate 21 × 21 km resolution [35].

#### Noise

Noise models were available only for Vancouver and Toronto and were therefore only included in sensitivity analyses. In Vancouver, the Computer Aided Noise Abatement (CadnaA) propagation model was used to derive residential noise estimates. Inputs to the model included traffic data (e.g., speed limits, traffic volume, fleet composition, and road width), railway data (e.g., type of train, velocity, and frequency), aircraft data and building heights and footprints. The creation and evaluation of this model has been described previously [36]. In Toronto, a LUR model was used to predict noise from 554 short-term (30 min) and 10 long-term (1 week long) measurements [37]. The Toronto noise LUR model explained 74% and 68% of the spatial variability in the hot and cold season, respectively.

#### Opportunity for physical activity

Park proximity and neighborhood walkability may link green space, mother’s physical activity and birth outcomes. We used a dichotomous variable for distance to park (residing less than or more than 400 m) since residents living closer to a park are more likely to use the park for physical activity [38, 39]. WalkScore^®^ was used to represent neighborhood walkability. The walkability measure is a proprietary measure that is based on the distance to nearby places, block length and pedestrian friendliness, ranging from 0 to 100, with higher values reflecting high walkable neighborhoods in which daily errands would not require a vehicle (WalkScore 2017).

#### Perceived social support

Social support was measured using the validated 4-item Interpersonal Support Evaluation List-12 (ISEL-12), a measure of perceived social support [40]. This survey was administered at 36 weeks of gestation. Example questions included: I feel that there is no one I can share my most private worries and fears with; if I were sick, I could easily find someone to help me with my daily chores (options (1) definitely false, (2) probably false, (3) probably true, (4) definitely true). An overall social support score was generated by summing the responses of all 12 items, with values ranging from 22 to 44 (higher score indicates higher perceived social support). This variable was included as a continuous variable in regression models, with tertiles examined in stratified analysis.

#### Psychological stress and depression

Participants answered questions about self-reported experienced stress using the Perceived Stress Scale (PSS) [41], which is a validated 10-item scale measuring the degree to which situations in one’s life are appraised as stressful. The values ranged from 0 to 40 with a higher score indicating more stress [41]. Similar to social support, perceived stress was reported around 36 weeks of pregnancy and was included as a continuous variable, with tertiles examined in stratified analysis.

Participants also answered questions regarding depression using the 20 item Center for Epidemiologic Studies Depression Scale Revised (CESD-R) [42]. The range of possible scores is between 0 and 60 with a higher number indicating more symptoms of depression. We used the 36 week CESD-R questionnaire to align with the other psychosocial measures. The depression measure was examined as a continuous variable and tertiles examined in stratified analysis.

### Covariates

A comprehensive set of covariates were available at the individual and neighborhood level to control for potential confounding factors. Individual socio-demographic covariates collected from the study questionnaires include: maternal age; mother and father’s education (coded as less than high school diploma, high school diploma, some college, college degree and post graduate degree); mother and father’s race (White, Asian, and Other); and household income ($0–$9999, $10,000–$19,999, $20,000–$29,999. $30,000–$39,999, $40,000–$49,999, $50,000–$59,999, $60,000–$79,999. $80,000–$99,999, $100,000–$149,999, > $150,000). Pregnancy data included parity (first birth or not); smoking during pregnancy (yes/no); and month and year of birth. An indoor air quality index was also included that estimates the total exposure to multiple oxidizing chemicals in the home, which provides a more complete assessment of multiple indoor exposures linked to inflammation [43].

Neighborhood SES variables and population density (per km^2^) were also included at the census tract level for the 2011 census and linked to each birth. Variables included percent white; percent adult population without a high school diploma; median household income; percentage of the population below the poverty line; and percent unemployment. Population density (coded into quintiles) was used to control for potential confounding factors associated with urban form, such as inner city cores or suburban areas. We also derived a proximity to water (e.g., river, lake, ocean) variable (residing less than or more than 500 m) to control for blue-space related influences that have been identified in other research [44].

### Statistical analysis

We evaluated the associations between mother’s residential green space exposure during pregnancy and term birth weight, and examined the following pathways: environmental exposures (NO_2_, PM_2.5_ and O_3_), physical activity (Walkscore ^®^, park and water proximity), psychosocial influences (social support), and psychological influences (maternal stress and depression).

First, we examined the overall association between green space and birth weight. Linear regression, with a random intercept to account for the clustering within each study city, was used to assess associations between residential NDVI and term birth weight (babies born ≥ 37 weeks of gestation) adjusting for a comprehensive set of individual and neighborhood confounding factors. The fully adjusted model includes all individual and neighborhood variables summarized previously. Associations between NDVI and birth weight are presented corresponding to a 0.1 unit increase in NDVI. We also ran overall models stratified by individual and contextual level variables selected a priori to examine potential effect modification and evaluate residual confounding.

Next, we examined hypothesized pathways using incremental analyses. We used linear regression to examine the associations between green space and NO_2_, PM_2.5_, O_3_, Walkscore^®^, park and water proximity, social support and maternal stress and depression. Incremental models were then run that included each hypothesized pathway variable(s) separately and then all pathways together. Each model was adjusted for potential confounding factors included in the overall adjusted model.

Several sensitivity analyses were conducted to examine the robustness of our models. We explored city-specific models as cities had large differences in terms of size/density, climate, and green space characteristics. Vancouver and Toronto also had available noise models and sensitivity analyses were conducted to examine how the NDVI and birth weight associations changed in these cities once noise exposures were included. We also evaluated the sensitivity of our results to including a random effects for neighborhoods, represented by Census Tract boundaries. And lastly, we examined the associations between season and birth weight by combining several months into a season variable (June–August, September–November, December–February, March–May).

## Results

**Fig. 1:**
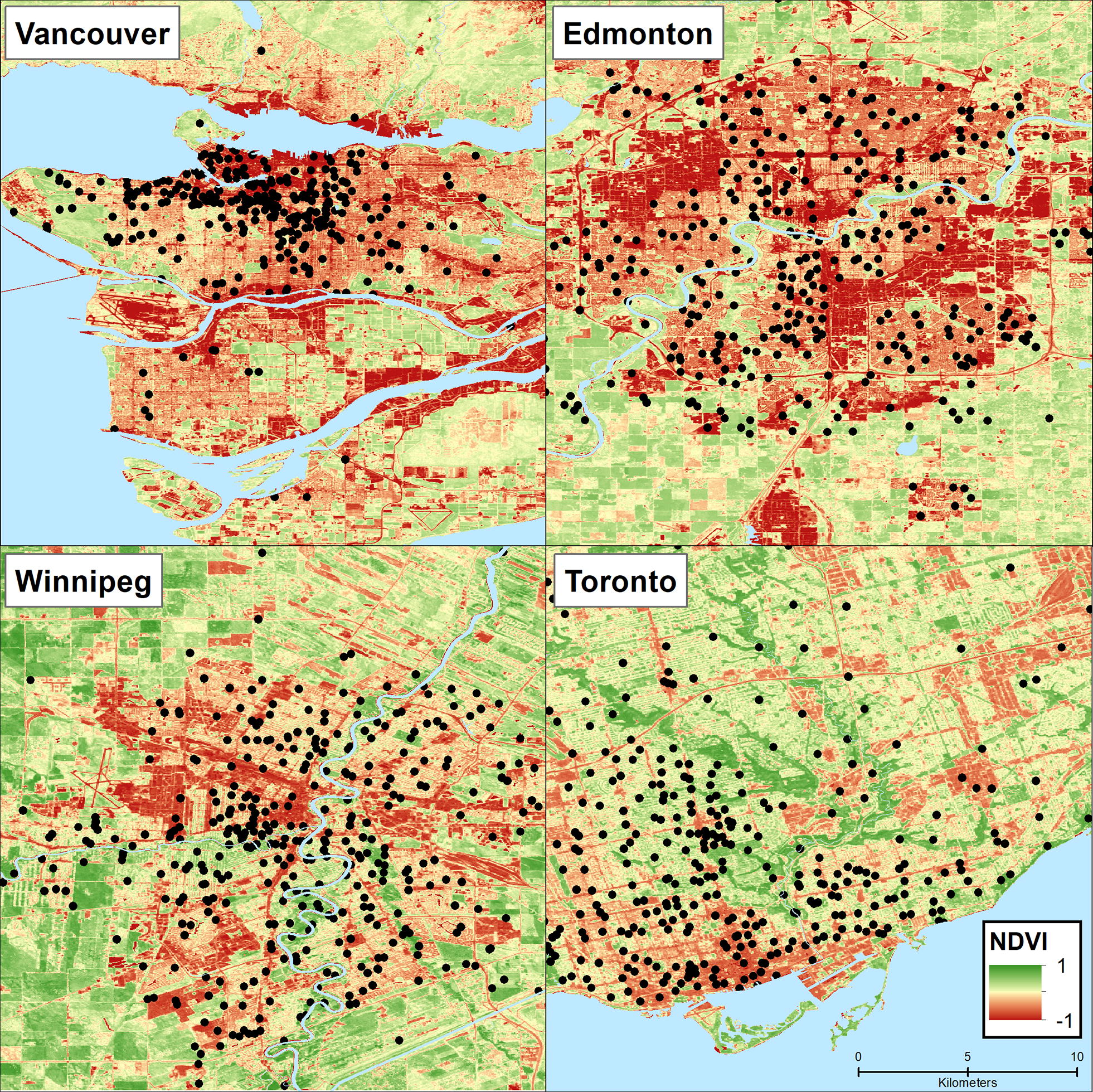
Map of CHILD participants (points randomly assigned within study Census tracts) in the four Canadian cities, with average NDVI levels for the year 2010

After exclusions for missing residential addresses and covariate information and restricting to urban metropolitan areas we examined 2510 births in the CHILD study. Figure 1 illustrates the general location of CHILD participants (locations randomly assigned to Census Tracts) by the four CHILD cities, with average NDVI levels for the year 2010.

Table 1 provides selected descriptive statistics for the 2510 births included in our analysis by quartiles of average NDVI within 500 m during pregnancy. The mean NDVI (500 m buffer) during pregnancy was 0.26 with a range of 0.05–0.61. Term birth weight was higher (3491 g) in quartile 4 (reflecting higher NDVI) compared to quartile 1 (3458 g). Differences in key socio-demographic characteristics by NDVI quartile were observed for race/ethnicity, education and income. Overall, the CHILD study is well educated (93.2% of mothers had at least some college education) and predominantly white (70.8%). Population density, NO_2_ and stress are lower in the highest green space quartile compared to the least green quartile, and the WalkScore^®^ measure is higher in the least green areas.

**Table 1:** Selected characteristics of 2510 births in the CHILD study by quartile of average NDVI within 500 m of mothers residences during pregnancy

	Entire cohort	Average NDVI (500 m Buffer)			
		Q1 < 0.21	Q2 0.21–0.25	Q3 0.26–0.30	Q4 > 0.30
Births (n)	2510	627	628	631	624
Birth weight (g) ($$ \bar{x}, sd $$)	3474 (498)	3458 (522)	3471 (481)	3475 (476)	3491 (513)
Maternal age ($$ \bar{x}, sd $$)	32.1 (4.6)	32.1 (4.5)	32.0 (4.5)	32.0 (4.8)	32.5 (4.4)
Maternal education (%)					
High school diploma	6.8	6.8	7.6	8.1	4.9
Some college	27.5	26.2	27.4	27.5	28.8
College degree	45.0	44.8	46.5	42.9	45.8
Post Graduate degree	20.6	22.1	18.5	21.5	20.5
Household income (%)					
< $30,000	2.8	4.1	2.1	2.4	2.9
$30,000–$49,999	4.9	4.4	5.5	5.6	3.6
$50,000–$79,000	21.3	19.7	21.5	25.5	18.7
> $80,000	71.0	71.8	70.9	66.5	74.7
Maternal ethnicity (%)					
White	70.8	68.8	70.1	71.9	72.4
Asian	10.7	11.0	12.0	9.1	10.5
Other	18.5	20.2	17.9	19.0	17.1
Smoked during pregnancy (%)	3.3	2.9	3.8	3.4	2.9
Moved during pregnancy (%)	11.5	15.2	10.8	8.5	11.8
Social support ($$ \bar{x}, sd $$)	31.3 (2.2)	31.2 (2.3)	31.4 (2.2)	31.3 (2.2)	31.0 (2.0)
Depression ($$ \bar{x}, sd $$)	9.5 (7.4)	9.7 (7.8)	9.6 (7.8)	9.6 (7.6)	9.2 (6.6)
Stress ($$ \bar{x}, sd $$)	12.5 (6.3)	12.8 (6.4)	11.9 (6.3)	12.6 (6.2)	12.6 (6.2)
Walk score ($$ \bar{x}, sd $$)	60.0 (27.7)	70.0 (28.7)	61.0 (27.6)	58.4 (24.7)	49.8 (25.5)
NO_2_ air pollution (ppb) ($$ \bar{x}, sd $$)	18.3 (5.1)	20.3 (5.3)	18.4 (4.5)	18.0 (4.6)	16.5 (5.1)
PM_2.5_ air pollution (ppb) ($$ \bar{x}, sd $$)	6.9 (1.5)	7.0 (1.4)	7.0 (1.3)	7.1 (1.4)	6.7 (1.6)
O_3_ air pollution (ppb) ($$ \bar{x}, sd $$)	25.1 (4.5)	25.0 (4.5)	25.0 (4.3)	25.3 (3.9)	25.7 (4.0)
Population/km^2^ ($$ \bar{x}, sd $$)	4370 (3406)	5789 (4580)	4614 (3230)	3775 (2396)	3280 (2413)
Neighborhood median household income ($$ \bar{x}, sd $$)	70,498 (29,597)	65,346 (26,899)	68,812 (26,867)	72,059 (27,060)	76,007 (35,887)
Neighborhood % minority ($$ \bar{x}, sd $$)	33.2 (23.0)	36.1 (21.1)	34.2 (22.7)	32.8 (24.0)	29.5 (23.7)
City (%)					
Vancouver	24.7	28.2	25.1	20.2	21.9
Edmonton	25.0	21.1	26.5	31.6	21.3
Winnipeg	23.4	23.2	24.0	22.7	24.9
Toronto	26.9	27.4	24.5	25.6	31.9

### Associations between green space and term birth weight

Table 2 summarizes model results for changes in term birth weight associated with a 0.1 increase in residential NDVI during pregnancy. We observed increased birth weight with increasing NDVI for buffer distances greater than 100 m, with the largest associations for NDVI within 500 m. Adjustment for individual and neighborhood covariates led to an attenuation of the association for NDVI within 100 m, but increased associations for NDVI in 500 and 1000 m.

**Table 2:** Changes in term birth weight associated with a 0.1 IQR increase in residential NDVI during pregnancy

NDVI Buffer	Model 1 β (95% CI)	Model 2 β (95% CI)	Model 3 β (95% CI)
100 m	14.1 (− 5.4, 33.7)	3.9 (− 17.4, 25.2)	4.0 (− 18.1, 26.1)
250 m	16.5 (− 4.6, 37.6)	11.6 (− 11.4, 34.6)	12.7 (− 11.6, 36.9)
500 m	18.8 (− 3.4, 41.1)	18.3 (− 6.1, 42.7)	21.5 (− 4.6, 47.7)
1000 m	15.7 (− 7.2, 38.7)	14.4 (− 10.8, 39.5)	17.8 (− 9.5, 45.0)

Model 1: Unadjusted model includes gestational age

Model 2: Model 1 plus individual characteristics: baby’s sex, year and month of birth, mother’s age, mothers smoking during pregnancy, mother/father education, mother/father race/ethnicity, household income, indoor air quality index and city

Model 3: Model 2 plus neighborhood household income, % without high school education, unemployment, % minority and population density at 1 km

**Fig. 2:**
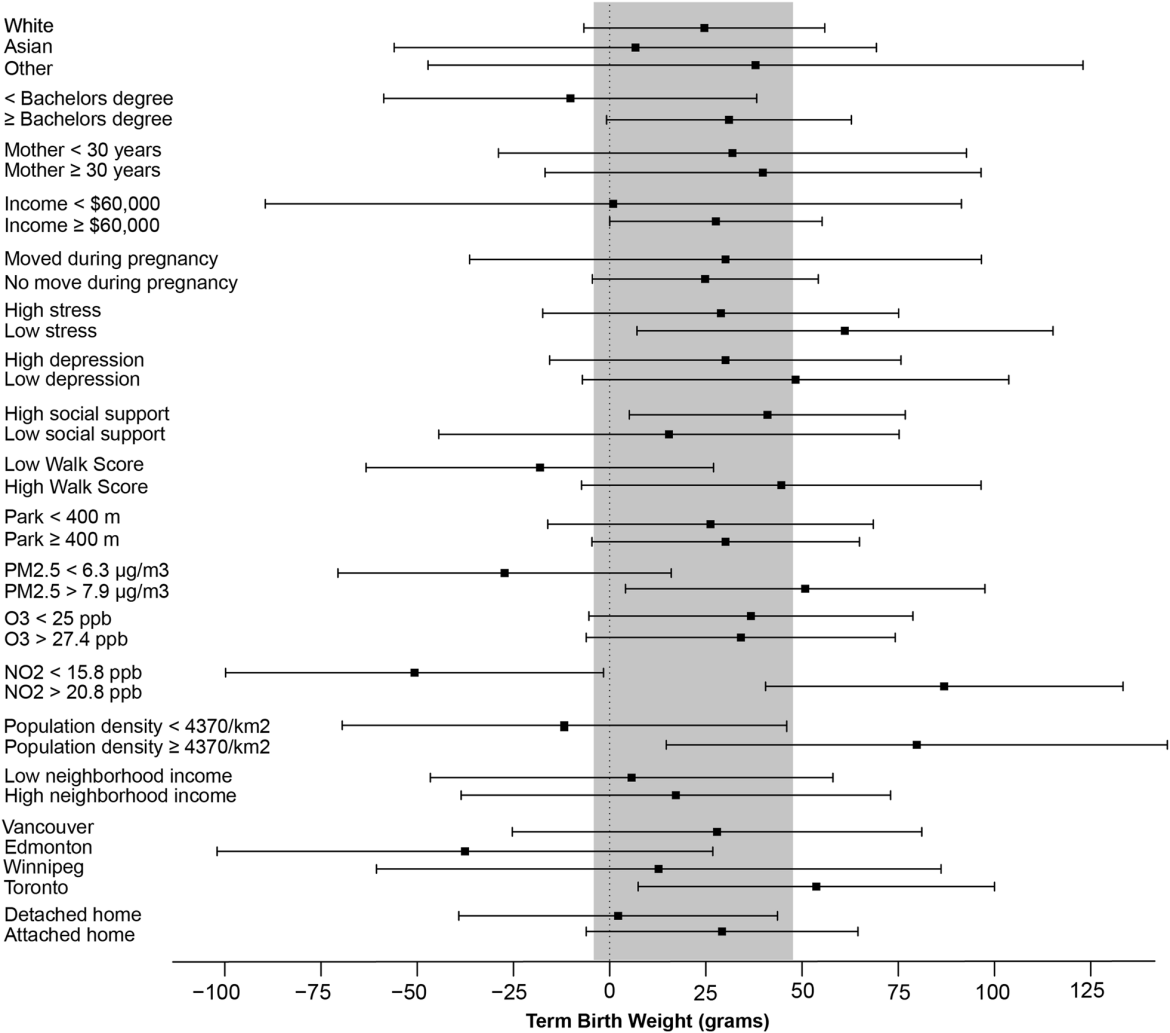
Stratified models of change in term birth weight (grams) for a 0.1 unit increase in average NDVI within 500 m of mothers’ homes during pregnancy. Shaded region represents 95% confidence intervals for overall model results

Figure 2 summarizes stratified models of green space and term birth weight. We summarize only NDVI within 500 m as this is the distance in which we observed the largest and most consistent associations with term weight. We see patterns of larger associations between NDVI and birth weight for higher SES mothers (higher educated, white and higher income neighborhoods) and mothers living in higher population density neighborhoods (represented by population density as well as higher NO_2_ and PM_2.5_ exposures and neighborhood walkability scores). Mothers with low perceived social support also had larger associations. Models stratified by CHILD cities demonstrated heterogeneous results, with large positive associations between NDVI and birthweight for Toronto and Vancouver and a negative association for Edmonton.

### Potential pathways linking green space and term birth weight

Incremental models of hypothesized pathway variables are summarized in Table 3. Data from the remaining buffer sizes demonstrated similar results to the NDVI 500 m exposure variable. These models are presented in general pathways (reducing environmental exposures; physical activity opportunities; psychosocial influences; psychological influences) with individual variables entered independently in models as well as all variables by pathway. As shown in Table 3, an increase in NDVI is significantly associated with a decrease in air pollution as hypothesized. The inverse association between walkability and park proximity may be reflecting more green space in sub-urban environments that are also associated with less walkability. An increase in NDVI was associated with a decrease in the distance to a park. No association was observed between green space and social support. Increasing NDVI was also associated with a decrease in depression but showed no association with stress. Overall, associations between term birth weight and residential green spaces tended to increase with each mediating pathway that was considered, except for physical activity opportunities, which slightly attenuated associations. The fully adjusted model, including model 3 plus all pathway variables showed an increase in birth weight of 29.3 g (95% CI − 1.2, 59.8) with a 0.1 increase in NDVI within 500 m.

**Table 3:** Associations between a 0.1 IQR increase in residential NDVI in 500 m and individual pathway variables and combined models of NDVI and term birth weight associations adjusted for pathway variables

	Association between NDVI (500 m) and each pathway variable*	Association between NDVI (500 m) and term birth weight adjusting for pathway variable**
Model 3 (fully adjusted model)		21.5 (− 4.6, 47.7)
Air pollution		
NO_2_ (ppb)	− 2.0 (− 2.3, − 1.8)	25.0 (− 2.5, 52.5)
PM_2.5_ (µg/m^3^)	− 0.2 (− 0.2, − 0.1)	21.7 (− 4.5, 47.8)
O_3_ (µg/m^3^)	− 0.1 (− 0.2, − 0.004)	24.4 (− 2.3, 51.2)
Model 3 + all air pollution variables		27.3 (− 1.0, 55.7)
Physical activity opportunities		
Walk score	− 7.7 (− 9.1, − 6.3)	19.0 (− 8.6, 46.6)
Park proximity (meters)	− 10.1 (− 212.1, 191.9)	16.1 (− 10.5, 42.7)
Water proximity (meters)	81.7 (− 123.1, 286.6)	21.8 (− 4.4, 48.0)
Model 3 + all physical activity variables		18.9 (− 8.8, 46.5)
Psychosocial influences		
Social support	− 0.1 (− 0.2, 0.4)	22.1 (− 5.1, 49.4)
Model 3 + social support		22.1 (− 5.1, 49.4)
Psychological influences		
Depression	− 0.4 (− 0.8, 0.1)	22.6 (− 4.6, 49.8)
Stress	0.0 (− 0.4, 0.4)	22.2 (− 5.1, 49.4)
Model 3 + All psychological influences variables		23.1 (− 4.3, 50.5)
Model 3 + all pathway variables		29.3 (− 1.2, 59.8)

* Associations are for a 0.1 IQR increase in residential NDVI in 500 meters

** Adjusted models include: gestational age, baby’s sex, year and month of birth, mother’s age, mothers smoking during pregnancy, mother/father education, mother/father race/ethnicity, household income, indoor air quality index, city, neighborhood household income, % without high school education, unemployment, % minority and population density at 1 km

### Sensitivity analyses

We evaluated the sensitivity of our main model results to the inclusion of noise estimates (for Vancouver and Toronto), neighborhood random effects, and season. Noise models were available for Vancouver and Toronto. Our main model for these two cities combined showed that a 0.1 unit increase in NDVI is associated with a 41.2 g (95% CI 7.8, 74.6) increase in term birth weight. This results reflects the larger associations we observed in our Vancouver and Toronto stratified models (Fig. 2). When all mediating variables (from Table 3) are added to the model for Vancouver and Toronto we observed a 60.2 g (95% CI 18.6, 101.8) increased for a 0.1 increase in NDVI. Further inclusion of noise estimates for these cities did not substantially attenuate the NDVI and term birth weight associations in our overall models. Including a random effect for census tracts, to further control for potential unmeasured contextual factors, did not change model results. We also explored including a variable for season in place of the birth month to determine if this would have an impact on birth weight, given the differences in season between the four cities in the CHILD study. In the overall model, we did not see any change in the associations once season was added. When including season in the models stratified by city the association was further attenuated in Winnipeg, with no changes in the other three cities.

## Discussion

We investigated the association and potential pathways linking proximity to green space during pregnancy (assessed using NDVI), and term birth weight for 2510 births in four Canadian cities. We observed positive associations between increasing exposure to green space and increased term birth weight in models adjusted for potential individual and neighborhood confounding factors. Overall associations for the four cities were not statistically significant, but stronger statistically significant associations were observed for mothers living in high-density locations and the cities of Vancouver and Toronto. We did not observe substantial reductions in the green space and birth weight association with the addition of hypothesized pathway variables, which was consistent across all cities.

Our overall findings of a potential association between residential green space and term birth weight (ranging from 0.4 to 22.1 grams with a 0.1 unit increase in NDVI) generally corresponds to the existing literature. In a separate study in Vancouver Canada, a 0.1 increase in NDVI (within 100 m of mothers homes) was associated with a 20.4 g increase in term birth weight [6]. If we restricted our analyses to Vancouver and to NDVI within 100 m we observed a 18.5 g (95% CI − 29.5, 65.9) increase per 0.1 increase in NDVI. While a number of other studies have observed increases in term birth weight with residential NDVI [4, 45], other studies have not [46]. For example, in Texas, there were strong positive impacts from greenness on birth weight in unadjusted models, but most of the relationship disappeared when including individual characteristics such as race and ethnicity [46]. In our study, we were able to control for several individual-level factors not available in registry based studies, and adjustments for individual socio-demographic factors tended to increase, rather than attenuate, our observed associations. This suggests that the associations we observed between green space and term birth weight are not due to residual confounding.

We observed mixed associations across the four Canadian cities included in the CHILD cohort. For Vancouver and Toronto, which are the third and first largest cities in Canada, respectively [47], we observed strong positive associations while there was a negative association in Edmonton and no association in Winnipeg. These differences may be due to a density related effect, as we observed stronger associations between green space and term birthweight for mothers living in locations with a population density > 4370 individuals per square km and for individuals with high NO_2_ exposure and walkability^®^ scores (typically located in high density areas). We note that in our stratified analyses, we found stronger association for mothers living in apartments/condos compared to those residing in single family detached house, another potential surrogate for density/urbanicity. The types of vegetation and associated biological exposures, the accessibility of green space, proximity to blue space and climate also varies considerably across these four cities, which could affect the nature of the association between green space and term birth weights. Toronto and Vancouver are more moderate climates (− 2 °C average minimum winter temp vs. − 16 °C in Edmonton and Winnipeg); however, our sensitivity analysis including season of birth did not change our findings. The results of our city specific analysis suggest that much more research is needed to understand if green space benefits are restricted to high-density city locations, where green space is limited, and how green space affects may vary across diverse climate conditions.

Understanding how green space may influence birth outcomes is needed and our study is the first to examine a comprehensive number of hypothesized pathways. Opposite to our a priori hypotheses, we found that the pathways examined (air pollution/noise, physical activity opportunities, social capital and stress/depression) did not explain the observed associations between green space and birth weight in these Canadian cities. This suggests that our variables may not be properly measuring the specific hypothesized pathways of interest; that other pathways not included in our models are present; or that the relationship between residential green space and birth outcomes is due to residual or unmeasured confounding factors.

In our analysis, controlling for NO_2_, PM_2.5_ and O_3_ did not change the association between NDVI and birth weight. There are mixed findings in the literature as to whether air pollution mediates green space effects and little is known specifically for adverse birth outcomes [2, 5, 32, 45]. For example, in a Spanish cohort, adjusting for NO_2_ reduced the magnitude of association between green space and birth weight from 22.3 to 17.1 g [5]. However, a similar study in Munich observed a slight increase in birth weight after adjusting for NO_2_ and PM_2.5_, from 12.2 to 16.2 and 14.8 g respectively [45]. A previous study in Vancouver, Canada examined the influences of air pollution and found little effect on the green space-birth weight associations [32]. The combined weight of these findings suggests that buffering of harmful environmental pollutants does not account for the observed associations between NDVI and birth weight; however, the relationships between home air pollution exposure and surrounding green space (and the relative locations of the green buffers) may require more detailed data to tease apart.

Examining the physical activity pathway, we found that Walkscore^®^ attenuated the association slightly (though not significantly), while proximity to parks and water had little effect. The correlation between NDVI and Walkscore^®^ was − 0.31. A growing body of research has demonstrated that physical activity during pregnancy can reduce the risk of having a low birth weight baby [16–19]. Unfortunately, we did not have information on mother’s physical activity levels during pregnancy and we only examined built environment features that may promote physical activity (and be related to green space). In the current analysis, the individual models stratified for walkability and distance to the nearest park has little effect on the green space association. In the future, a direct measure of physical activity (e.g., accelerometry) would be more appropriate for examining this pathway.

We observed a negative association between NDVI and mothers who reported depression but not stress. These findings are consistent with a study examining the association between NDVI and stress and depression in twins that found that greater access to green space was associated with less depression but found less evidence for effects on stress [48]. Maternal depression has also been linked to low birth weight [29, 49]. Consistent with this, the stratified analysis showed that green space had a larger positive effect on birth weight when the mother was not depressed, compared to depressed. A study in Bradford, England, also observed an association between green space and depressive symptoms in pregnant women but this was only significant for women with lower education or who were physically active [10]. The study also determined that physical activity was not a mediator of the relationship between green space and depressive symptoms [10]. Importantly, we were unable to specifically measure attention restoration, a hypothesized influence of green space [8]. In addition, our measure of stress may not have been sensitive to the hypothesized stress reduction potential of green space [8].

Social support has long been considered an important health indicator for women, with research showing that low social support has a significant direct effect on birth weight [27, 28]. In our study we observed that NDVI was weakly inversely correlated with social support. However, other studies have found that proximity to green space is associated with increased social support [26], by providing a meeting place for people to develop and maintain social interactions [25]. While our analysis did not show any changes in birth weight when adjusting for social support, in stratified models we observed a greater increase in birth weight for low compared to high social support (e.g., an increase of 41.0 g (95% CI .1, 76.8) for a 0.1 unit increase in NDVI within 500 m for mothers with reported low social support. This interaction is consistent with the literature demonstrating that health benefits from green spaces are unequally distributed among various SES strata. Similarly, Markevych et al. showed better outcomes among the less educated participants, so it could be assumed that a similar gradient of effect from green spaces applies to not only various SES strata but also to psychosocially different groups [45].

## Limitations

While this analysis contains substantial strengths (i.e., large population sample, four distinct cities spanning a large and diverse geographical area, and detailed individual and neighborhood confounding and mediating variables) and contributes important new information on the associations between green space exposure during pregnancy and term birth weight there are limitations to highlight. First, we used a satellite-derived green space measure (NDVI) around mothers’ residential addresses to assess “exposure” to green space. However, NDVI represents only the presence of green vegetation and does not capture type, quality or use of green space. Nevertheless, we have improved on previous studies by using 30 m NDVI estimates and averages for each 9 month pregnancy period, rather than summer maximum or yearly averages. Second, the variables we had available to measure our proposed pathways are not ideal in all cases (e.g., the lack of physical activity measures for mothers) and likely incorporate measurement error (e.g., air pollution and noise models). However, this is the first study that has attempted to evaluate such a diverse range of pathways linking green space to birth outcomes and the lack of attenuation in our green space estimates by these pathways cannot be explained by measurement error alone. Third, the mothers in the CHILD cohort are predominantly white, well educated women that have access to universal health care. This limited the variation in several key socio-demographic characteristics that have been shown previously to modify and potentially mediate green space associations with adverse birth outcomes. However, this also reduced the potential that our results are due to residual confounding by SES, a major question in the green space literature. Finally, even though this is a relatively large prospective birth cohort with very detailed data collection, sample size was limited for stratified and pathway analyses.

## Conclusions

We examined the relationship between green space and term birth weight in a large Canadian birth cohort spanning four cities. We observed positive associations between increasing exposure to green space and increased term birth weight in models adjusted for potential individual and neighborhood confounding factors. Overall associations for the four cities were not statistically significant, but stronger statistically significant associations were observed for mothers living in high-density locations and the cities of Vancouver and Toronto. The hypothesized pathways tested (the reduction of harmful environmental exposures; space for increased utilitarian and recreational physical activity; positive psychosocial influences; and reduced psychological stress and depression) did not explain these positive associations. Our results highlight the need to characterize the interactions between green space, urban density and climate related factors and further evaluate pathways linking residential green space exposures to improved birth outcomes.

## Abbreviations

AllerGen: Allergy, Genes and Environment
CadnaA: Computer Aided Noise Abatement
CESD-R: Center for Epidemiologic Studies Depression Scale Revised
CHILD: Canadian Healthy Infant Longitudinal Development
CHRONOS: Canadian and Hemispheric Regional Ozone and NOx System
CIHR: Canadian Institutes of Health Research
ISEL-12: Interpersonal Support Evaluation List-12
LUR: Land use regression
NCE: Network of Centres of Excellence
NDVI: Normalized difference vegetation index
NO_2_: Nitrogen dioxide
O_3_: Ozone
PM2.5: Fine particulate matter
PSS: Perceived Stress Scale
SES: Socioeconomic status
